# Supplementary material for: Three-Dimensional Bioprinting of an In Vitro Lung Model
Source: Int J Mol Sci. 2023 Mar 19;24(6):5852. doi: 10.3390/ijms24065852 (PMC10059924; doi:10.3390/ijms24065852)
Supplement: Supplementary file 1 [file ijms-24-05852-s001.zip › ijms-2233331-supplementary.pdf]

Supplementary Figures

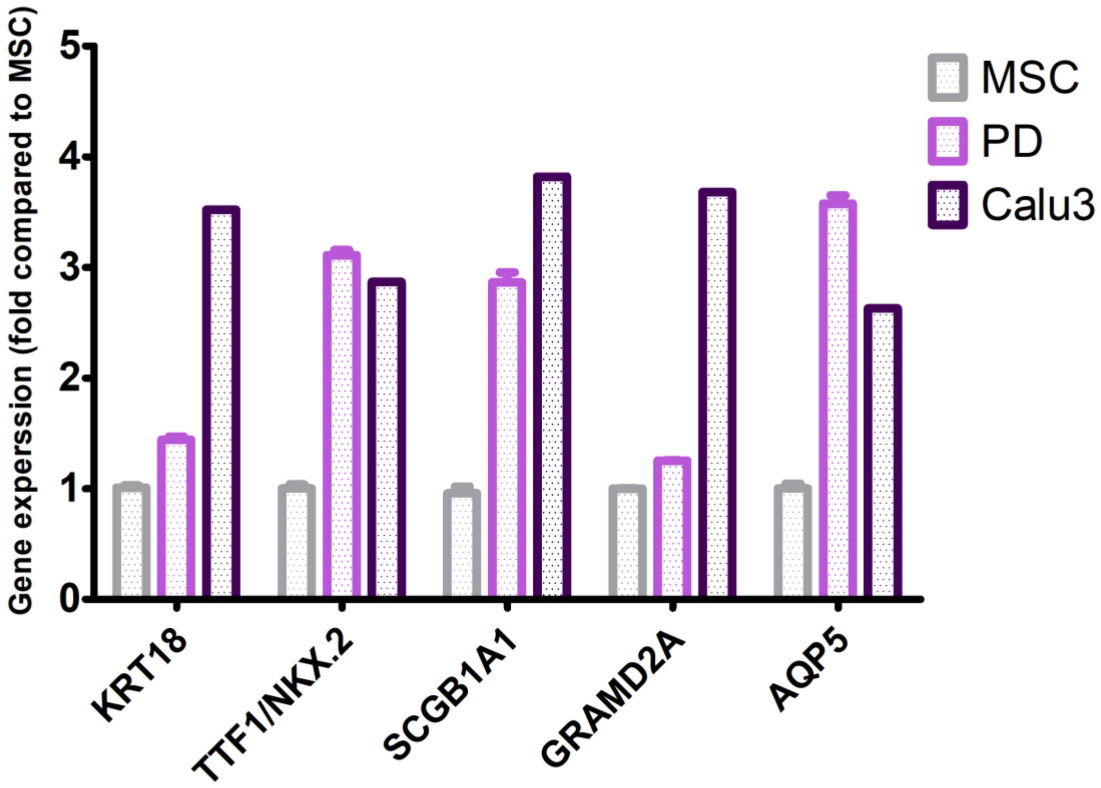

Supplementary Figure S1. Histogram of Gene Expression. Quantification of the cDNA band by ImageJ. Each target gene was normalized, with Beta-Actin being the reference gene. Fold comparing differentiated pulmonary cells (PD) and Lung adenocarcinoma cells (Calu-3) as a positive control with mesenchymal stem cells (MSC), undifferentiated cells. Statistical analyzes were performed using the GraphPad Prism 5 software. Fold Compared. Pulmonary genes: KRT18, TTF1/NKX.2, SCGB1A1,

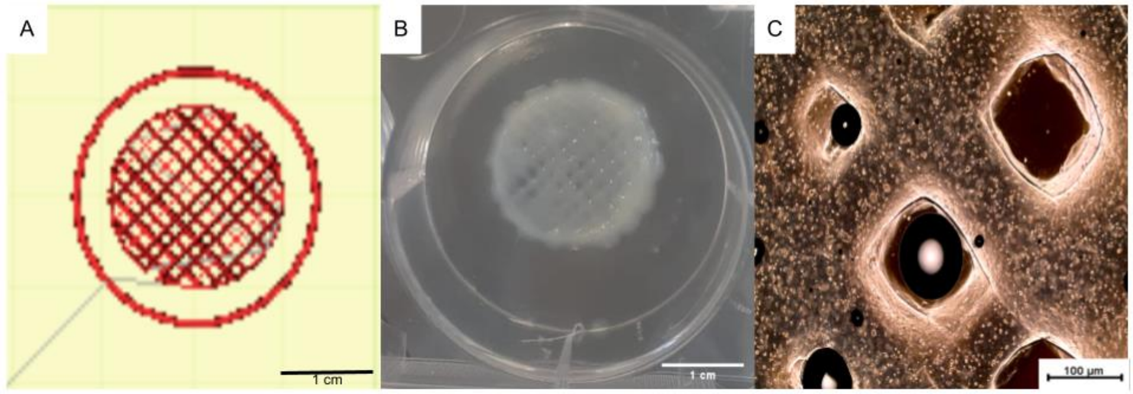

Supplementary Figure S2. 3D bioprinting. (A) Model used for bioprinting; (B) Bioprinted 3D structure; (C) Microscopy of the 3D structure.

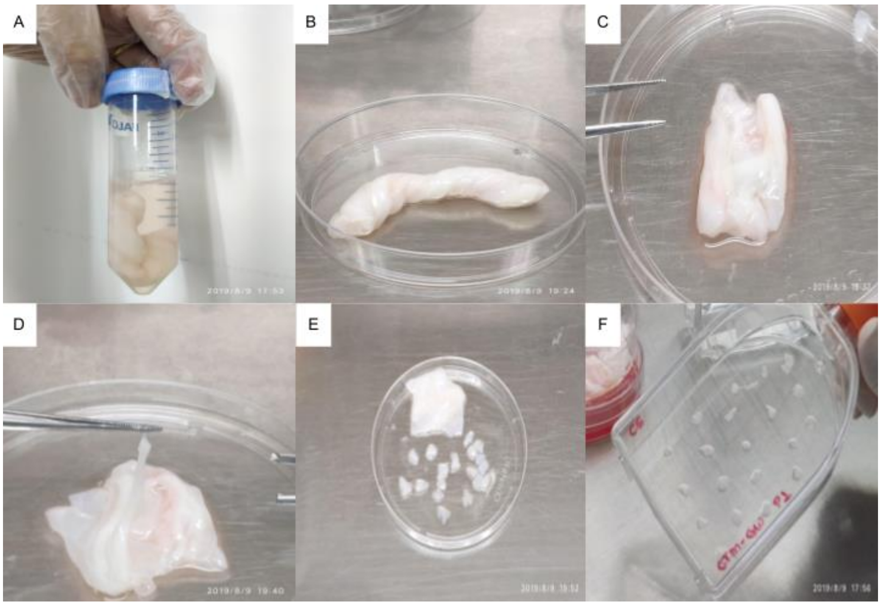

Supplementary Figure S3. Isolation of WJ-MSC. (A) Umbilical cord storage; (B) Fragment of the umbilical cord; (C) Longitudinal cut; (D) Removal of blood vessels; (E) Fragmentation of the umbilical cord; (F) Fragments a`rranged in the culture flask.
